# Biological specificity of CDK4/6 inhibitors: dose response relationship, *in vivo* signaling, and composite response signature

**DOI:** 10.18632/oncotarget.18435

**Published:** 2017-06-10

**Authors:** Erik S. Knudsen, Jack Hutcheson, Paris Vail, Agnieszka K. Witkiewicz

**Affiliations:** ^1^ University of Arizona Cancer Center, Tucson, AZ, USA; ^2^ Department of Medicine, University of Arizona, Tucson, AZ, USA; ^3^ Department of Pathology, University of Arizona, Tucson, AZ, USA; ^4^ Tri-Service Research Laboratory, San Antonio, TX, USA

**Keywords:** CDK4, palbociclib, abemaciclib, breast cancer, E2F

## Abstract

Recently developed potent and selective CDK4/6 inhibitors fall into two classes based on structure and toxicity profiles in clinical studies. One class, exemplified by palbociclib and ribociclib, exhibits neutropenia as a dose-limiting toxicity and requires discontinuous dosing. In contrast, the structurally distinct CDK4/6 inhibitor abemaciclib is dosed continuously, and has diarrhea and fatigue as dose-limiting toxicities. In preclinical models, palbociclib has been extensively studied and induces cell cycle inhibition in an RB-dependent manner. Thus far, abemaciclib has been less extensively evaluated. We found that abemaciclib cell cycle inhibitory activity is RB-dependent at clinically achievable concentrations. Abemaciclib elicited potent suppression of RB/E2F regulated genes associated with prognosis in ER-positive breast cancer. However, unlike palbociclib, at 250nM-1 µM doses abemaciclib induced cell death in RB-deficient cell lines. This response was associated with a rapidly-induced multi-vacuolar phenotype indicative of lysosomal membrane permeabilization that could be ameliorated with chloroquine. This event was not a reflection of inhibition of other CDK family members, but could be recapitulated with CBX4945 that inhibits casein and DYRK/HIPK kinases. To determine if these “off-target” features of abemaciclib were observed *in vivo*, mice harboring matched RB-positive and negative xenografts were treated with palbociclib and abemaciclib. *In vivo*, all of the apparent activity of abemaciclib was RB-dependent and strongly elicited suppression of cell cycle regulatory genes in a fashion markedly similar to palbociclib. Using gene expression data from cell lines and tumors treated with abemaciclib and palbociclib a composite signature of response to CDK4/6 inhibition was developed that included many genes that are individually required for tumor cell proliferation or viability. These data indicate that while abemaciclib and palbociclib can exert distinct biological and molecular effects, there are common gene expression features that could be broadly utilized in measuring the response to CDK4/6 inhibition.

## INTRODUCTION

CDK4/6 inhibitory agents have emerged as an important class of therapeutics that are currently tested for efficacy in a myriad of tumor types [1–4]. The CDK4/6 inhibitors in advanced clinical testing fall into two broad categories based on toxicity-prolife and dose-delivery schedules [1–4]. Palbociclib and ribociclib induce myelosuppression, and are generally dosed with a one-week break to recover neutrophil counts in patients [5, 6]. In contrast, structurally distinct abemaciclib is dosed continuously and elicits fatigue and diarrhea as potential dose-limiting toxicities [7]. The differences in side effects have lead to questions related to the selectivity of these kinases inhibitors and their respective mechanisms of action.

Palbociclib and ribociclib are based off of a similar pyrido [2,3-d]pyrimidin-7-one scaffold that was optimized for selectivity toward CDK4/6 [1, 8–10]. These inhibitors suppress both CDK4 and CDK6 kinase activity in the low nanomolar range. Preclinical studies demonstrated that the activity of these compounds is dependent on the presence of the RB tumor suppressor and effects on cell cycle progression [4, 11]. Consonantly, there is significant data suggesting that the only meaningful action of these drugs is CDK4/6 inhibition and cell cycle arrest [1].

Abemaciclib was developed from a 2-anilino-2,4-pyrimidine-[5-benzimidazole] scaffold [12]. The agent has potent activity against CDK4 and CDK6 and has been shown to harbor potent activity in early-phase clinical studies [7]. However, abemaciclib can also inhibit multiple other kinases *in vitro* at concentrations less than 100 nM [12, 13]. The extent to which these off-target events are relevant remains poorly understood. At present preclinical studies of abemaciclib are relatively limited compared to other CDK4/6 inhibitors [1]. Here, we addressed the biological relationship between palbociclib and abemaciclib to define specificity and relative on-target versus off-target effects in preclinical breast cancer models. These data were then utilized to develop a classifier of response to CDK4/6 inhibition that is applicable to these structurally diverse agents and should have broad applicability.

## RESULTS

**Figure 1:**
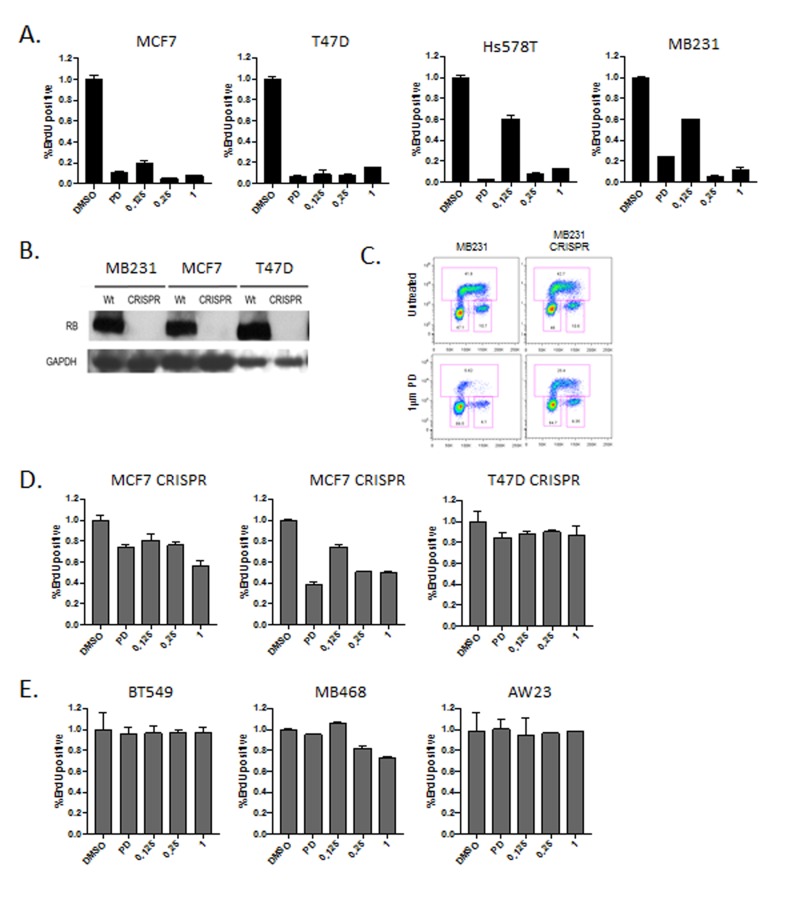
RB-dependent cell cycle inhibitory activity **A**. The indicated cell lines were treated with 1 µM palbociclib (PD) or 125 nM, 250 nM or 1 µM abemaciclib (LY). The relative BrdU incorporation was determined at 48 hours post-treatment. **B**. Immunoblots from the indicated cell lines developed with CRISP/Cas9 mediated deletion of RB. GAPDH is shown as a loading control. **C**. Representative BrdU (y-axis) vs. propidium iodide (x-axis) flow cytometry for RB-proficient and deficient models treated with palbociclib. **D**. The indicated cell lines were treated deleted for RB were treated with 1 µM palbociclib (PD) or 125 nM, 250 nM or 1 µM abemaciclib (LY). The relative BrdU incorporation was determined at 48 hours post-treatment. **E**. The indicated cell lines which are RB-deficient triple negative breast cancer models were were treated with 1 µM palbociclib (PD) or 125 nM, 250 nM or 1 µM abemaciclib (LY). The relative BrdU incorporation was determined at 48 hours post-treatment.

To define the response to abemaciclib in models of breast cancer we initially compared the cell cycle inhibitory effect of abemaciclib at a range of doses (LY: 125 nM - 1 µM) versus a constant dose of palbociclib (PD: 1 µM) (Figure 1A). Across luminal models (MCF7 and T47D) and triple negative models (Hs578T and MB231) there was a significant arrest of cell cycle at all doses of abemaciclib as evaluated by BrdU incorporation (Figure 1A). In general, a 250 nM dose of abemaciclib induced cell cycle inhibition comparable to 1 µM palbociclib dose. Cell cycle arrest occurred largely in the G1 phase of the cell cycle in a fashion that was consistent between palbociclib and abemaciclib (not shown). To determine if cell cycle inhibition was dependent on the presence of RB, gene editing was employed to develop matched RB gene ablated models (Figure 1B). Deletion of RB was associated with marked reduction in sensitivity to palbociclib. However, as previously reported using knockdown approaches, RB loss does not completely render models resistant to CDK4/6 inhibition (Figure 1C and 1D) [11, 14]. The requirement for RB was also observed with abemaciclib treatment in these matched models. Additionally, cell lines intrinsically lacking RB (AW23, MB468, and BT549) were equivalently resistant to the cell cycle inhibitory effects of both palbociclib and abemaciclib (Figure 1E). These data suggest that the RB-pathway is required for the cell cycle inhibitory activity of these CDK4/6 inhibitors.

**Figure 2:**
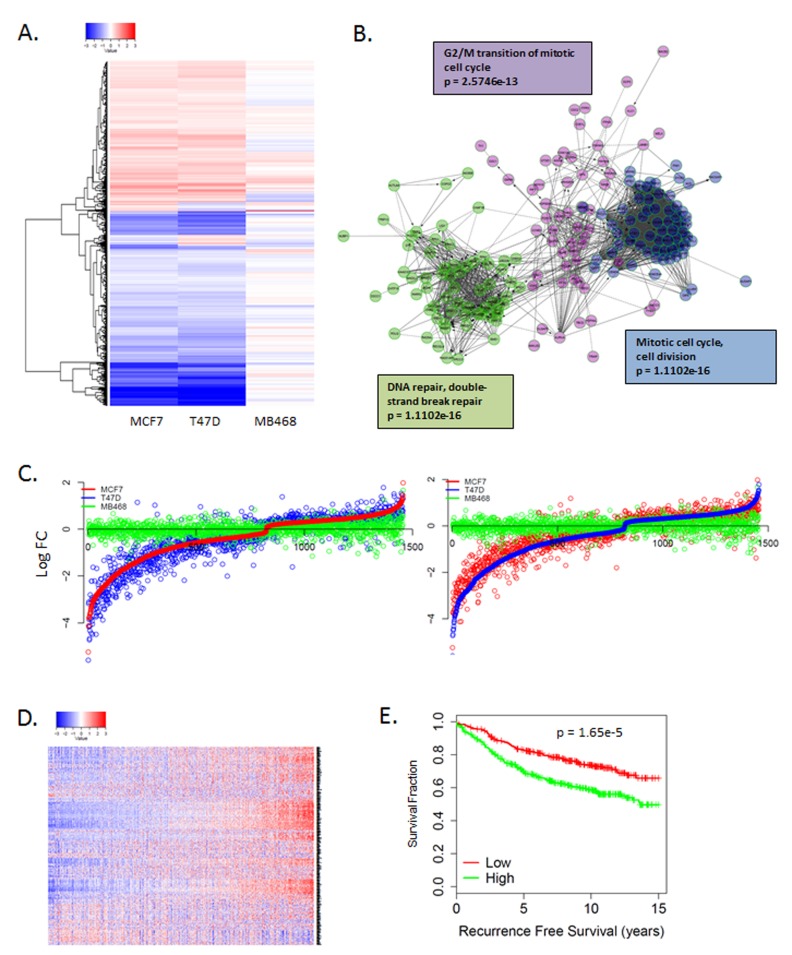
Unbiased gene expression response to CDK4/6 inhibition **A**. Heatmap illustrating gene expression changes that occurred in MCF7 or T47D vs. MB468 cells with 250 nM abemaciclib. Data are from triplicate experiments. **B**. Network analysis of genes repressed by abemaciclib demonstrating key enrichment of genes regulating cell cycle transitions. **C**. Comparison of gene expression changes in MCF7, T47D, and MB468 cells. Log fold change is plotted on the y-axis with a given gene indicated by the dot. **D**. Heatmap showing the coordinate expression of abemaciclib repressed genes in a collection of 967 ER+ breast cancers. **E**. Kaplan-meier curve shows the association of abemaciclib repressed genes with recurrence-free survival (*n* = 736).

To further explore the mechanism of action, gene expression analysis was performed on MCF7 and T47D cells that were treated with 250 nM abemaciclib and the RB-deficient MB468 cell line served as an RB-deficient control. In general abemaciclib and palbociclib demonstrated similar impact on gene expression in RB-proficient models that were absent in RB-deficient models (Figure 2A, Supplementary Figure 1). Since RB functions as a transcriptional co-repressor to elicit biological function [15–17], we focused on genes repressed by CDK4/6 inhibitors. Analysis of repressed genes demonstrated significant attenuation of the E2F-transcription factor regulated genes associated with cell cycle progression (Figure 2B, Supplementary Figure 1) [18]. While there were specific genes induced upon abemaciclib treatment, these alterations were variable across utilized models and did not conform to distinct enrichment by gene ontology (Supplementary Figure 1). The gene repressive response was highly conserved between MCF7 and T47D cells (Figure 2C, Supplementary Figure 1). The abemaciclib repressed genes were associated with prognosis in ER-positive breast cancer (Figure 2D), similar to previously reported prognostic impact of palbociclib regulated genes [18]. Overall, there is a significant concordance between the response to palbociclib (1 µM) and abemaciclib (250 nM) transcriptionally (Supplementary Figure 1).

**Figure 3:**
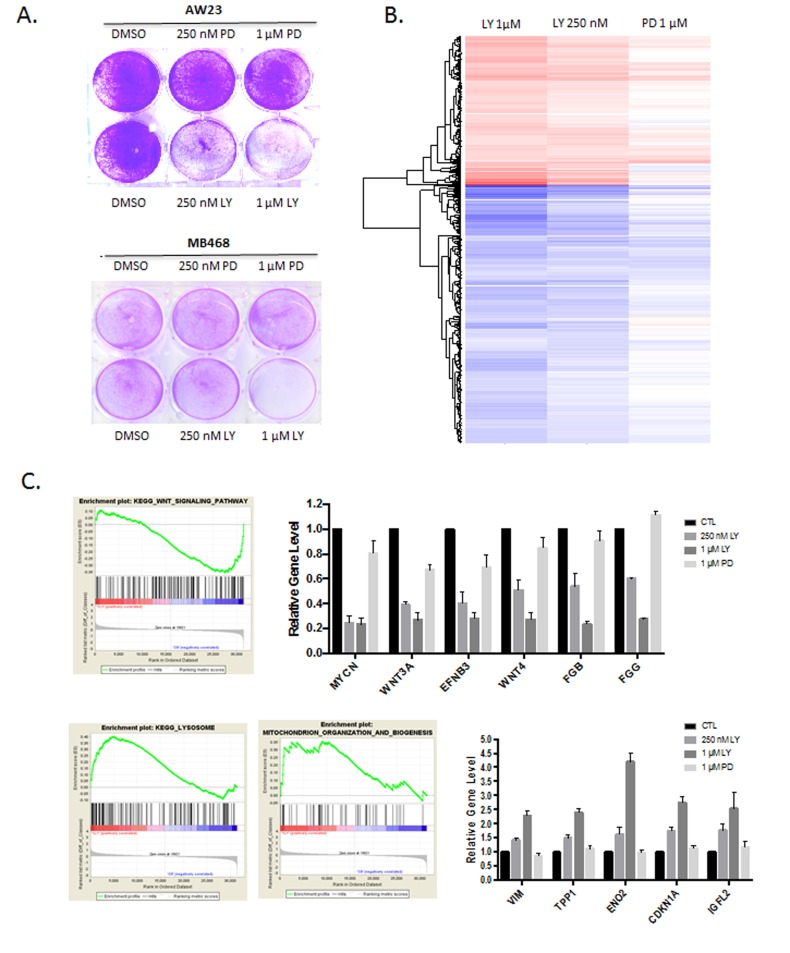
RB-independent activities of abemaciclib **A**. Crystal violet staining of the indicated cell lines treated with palbociclib (PD) or abemaciclib (LY). Representative images are shown. **B**. Significant gene expression changes in MB468 cells are shown in the heatmap. **C**. Gene set enrichment analysis and expression of select induced genes with abemaciclib treatment are shown.

In spite of the general RB-dependent response to abemaciclib in terms of cell cycle inhibition, we observed that at doses of 250-1000 nM abemaciclib impacted on viability of RB-negative models. In contrast, the RB-deficient triple negative cell lines AW23 and MB468 were resistant to the effect of palbociclib on cell survival (Figure 3A). To evaluate the potential mechanism through which the cell death could be mediated, gene expression alterations were analyzed in the RB-negative MB468 cells treated with abemaciclib (at 250 nM and 1 µM doses) and palbociclib (1 µM dose). These data revealed significant alterations in transcript levels that were specific to abemaciclib. (Figure 3B, Supplementary Figure 2). The repressed genes were not highly enriched by gene ontology analysis (Supplementary Figure 2); however, by gene set enrichment analysis we observed repression of multiple genes associated with WNT-signaling (Figure 3C). Interestingly, WNT-signaling is dependent on casein kinase activity in triple negative breast cancer models including MB468 cells, and abemaciclib can inhibit purified casein kinases in the nanomolar range [12, 19]. There was also induction of multiple genes involved in important biological functions such as lysosome biogenesis, mitochondria organization, and stress response (Figure 3C, Supplementary Figure 2).

**Figure 4:**
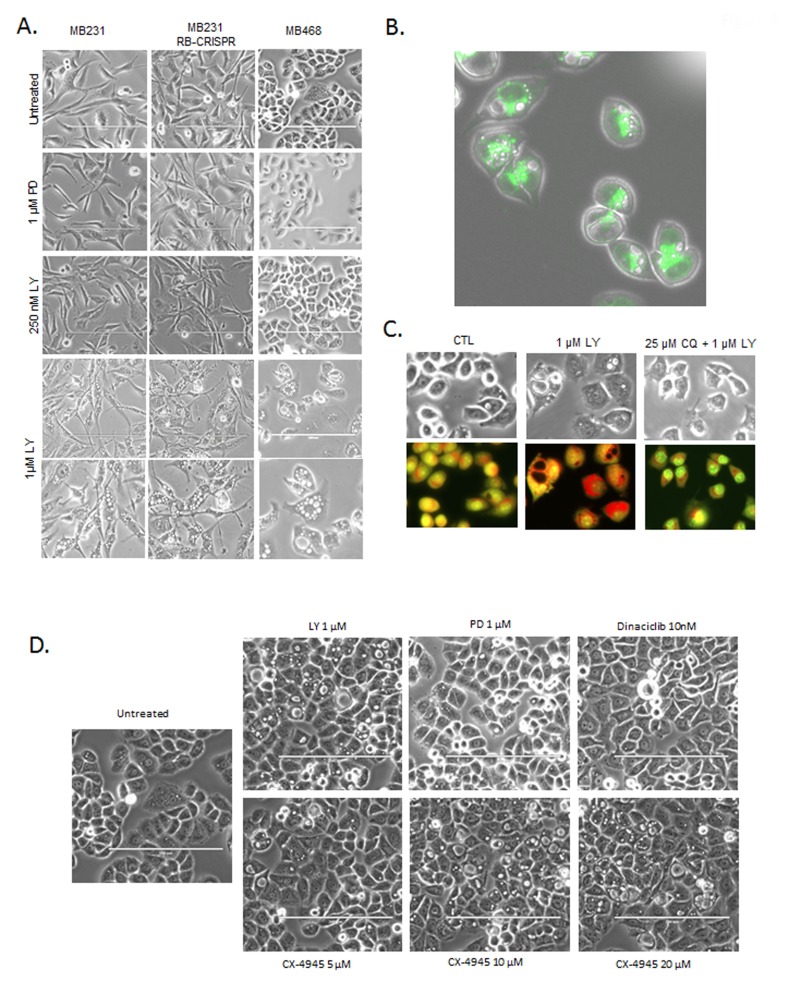
Defining RB-independent impacts of abemaciclib and cytotoxicity **A**. The indicated cell lines were treated with the indicated doses of palbociclib (PD) and abemaciclib (LY). Representative phase contrast micrographs are shown, with the bottom row showing further magnification of the 1 µM abemaciclib treated field. **B**. Lysotracker green was used to localize lysosomes relative to the vacuolar structures. Representative image of MB468 cells treated with 1 µM abemaciclib are shown, merged with the phase contrast image. **C**. MB468 cells were treated with the indicated agents and stained with acridine orange after 6 hours of treatment. Red staining-denotes the lysomal accumulation. **D**. Phase contrast images of cells treated with the indicated agents.

Evaluation of cellular morphology was used to infer potential cause of reduced cell viability. MB231 and MB468 cells treated with increasing doses of abemaciclib, displayed striking multi-vacuolar phenotype and at 1 µM dose (Figure 4A). This phenotype was independent of RB, as the CRISPR/CAS9 RB deleted models exhibited similar phenotype (Figure 4A). Interestingly, this phenotype was observed across multiple cell types, but not in every cell line evaluated, and was apparent within 6 hours (not shown). The presence of multiple large vacuoles along this time-frame of exposure is indicative lysosomal dysfunction [20]. Using lysotracker, we observed lysosomes juxtaposed to the vacuolar structures (Figure 4B). Cells treated at short time points (6 hours) with abemaciclib also exhibited acridine orange acidification, which was attenuated by chloroquine, further supporting notion that lysosomes were associated with vacuolar structures (Figure 4C). In order to decipher the potential basis of this effect, a number of agents that inhibit kinases suppressed by abemaciclib were applied to cells. As shown, palbociclib did not induce the multi-vacuolar phenotype, nor did the pan-CDK inhibitor dinaciclib (Figure 4D). The agent CX-4945 inhibits casein, DYRK, and HIPK kinases similar to the off-target spectrum of abemaciclib activity [12, 13]. Treatment with CX-4945 induced multiple vacuoles consistent with previously published studies [13] (Figure 4D). These data suggest that at higher concentrations (>250 nM) in cell based assays, off-target effects of abemaciclib that are non-CDK mediated are likely responsible for cytotoxicity and harbor effects related to lysosome integrity. These data provide a strong indication that abemaciclib has activities distinct from palbociclib and can elicit a cytotoxic phenotype that is independent from RB.

**Figure 5:**
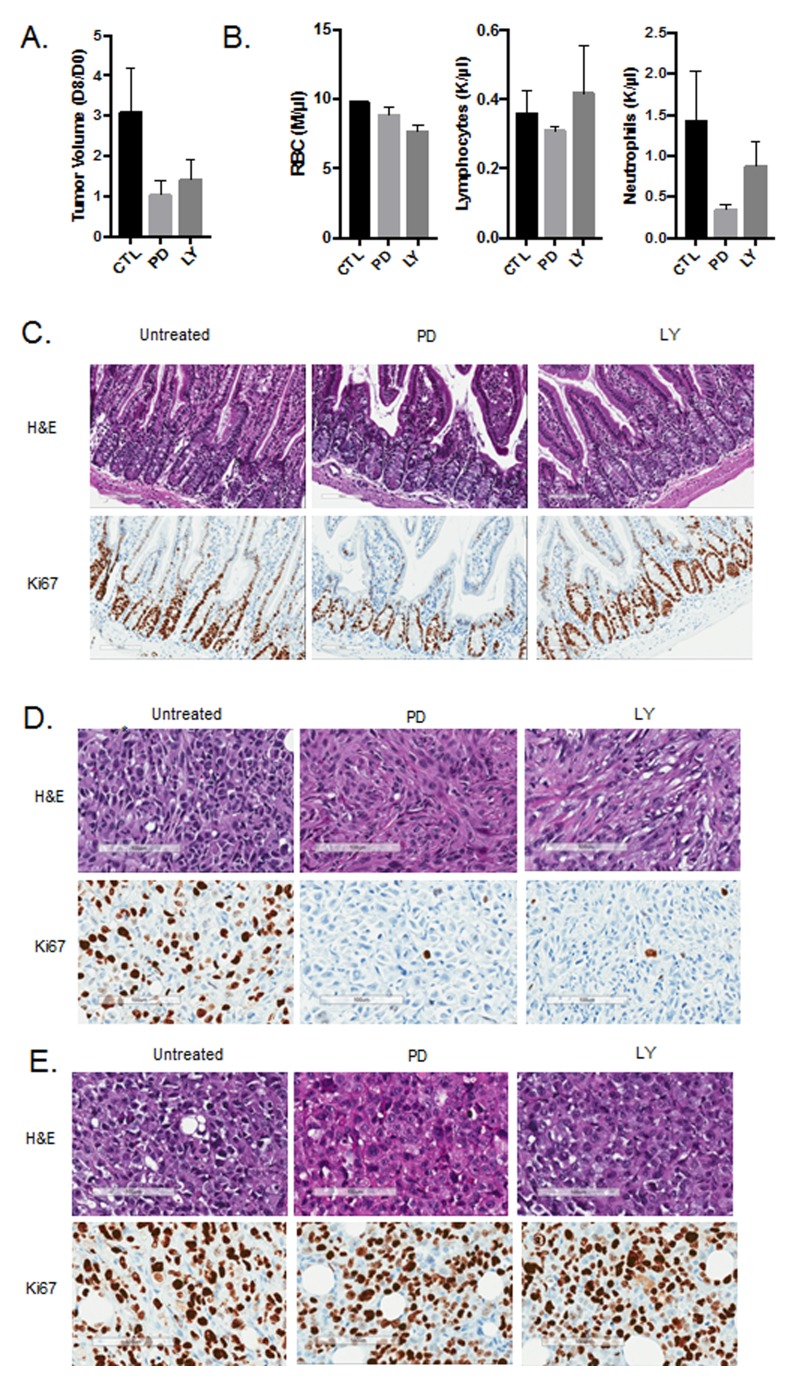
*In vivo* activity of CDK4/6 inhibitors **A**.Tumor volume was determined at day 0 of treatment and after 8 days of treatment. The ratio in tumor volume is shown, both abemaciclib (LY) and palbociclib (PD) significantly inhibited tumor growth (*p < 0.05). **B**. Complete blood counts were performed on day 8 of treatment and the total red blood cell (RBC), neutrophil, and lymphocyte counts are shown. Both abemaciclib and palbociclib treatment lead to reduced neutrophil counts. **C**. Immunohistochemical staining of small intestine from mice treated with the indicated agents. Ki67 staining is shown. **D**. Immunohistochemical staining of MB231 tumors from mice treated with the indicated agents. Ki67 staining is shown. **E.** Immunohistochemical staining of MB231 RB CRISPR/CAS9 deleted tumors from mice treated with the indicated agents. Ki67 staining is shown.

One of the key questions from the *in vitro* analysis is the extent to which these observations are relevant *in vivo*. To address this point, MB231 cells with intact or CRISPR/CAS9 deleted RB were injected into the mammary fat pad of NOD-SCID mice. Tumors were allowed to grow to approximately 500 mm^3^ and treated with either palbociclib (125 mg/kg) or abemaciclib (100 mg/kg) daily by oral gavage for eight days. Treatment with both palbociclib and abemaciclib resulted in the inhibition of tumor growth in RB-proficient tumors (Figure 5A). To delineate the impact related to hematological toxicity, complete blood counts were performed on the mice. The absolute counts of red blood cells and lymphocytes were not influenced by palbociclib or abemaciclib treatment (Figure 5B). However, neutrophil counts were diminished with both CDK4/6 inhibitors, albeit palbociclib had a more significant impact (Figure 5B). These data suggest that the mouse models reflect toxicities observed in patients. The impact of both agents on the mouse intestine was also evaluated. While both agents had an effect on the proliferation of intestinal stem cells, abemaciclib had a more modest effect and there was no induction of the vacuolar phenotype as observed in cell culture (Figure 5C). Both CDK4/6 inhibitors potently restricted the proliferation of tumors as indicated by the suppression of Ki67 (Figure 5D), which was mediated in an RB-dependent fashion (Figure 5E). Together, these data suggest that the predominant impact of palbociclib and abemaciclib was occurring through classical RB-dependent mechanisms.

**Figure 6:**
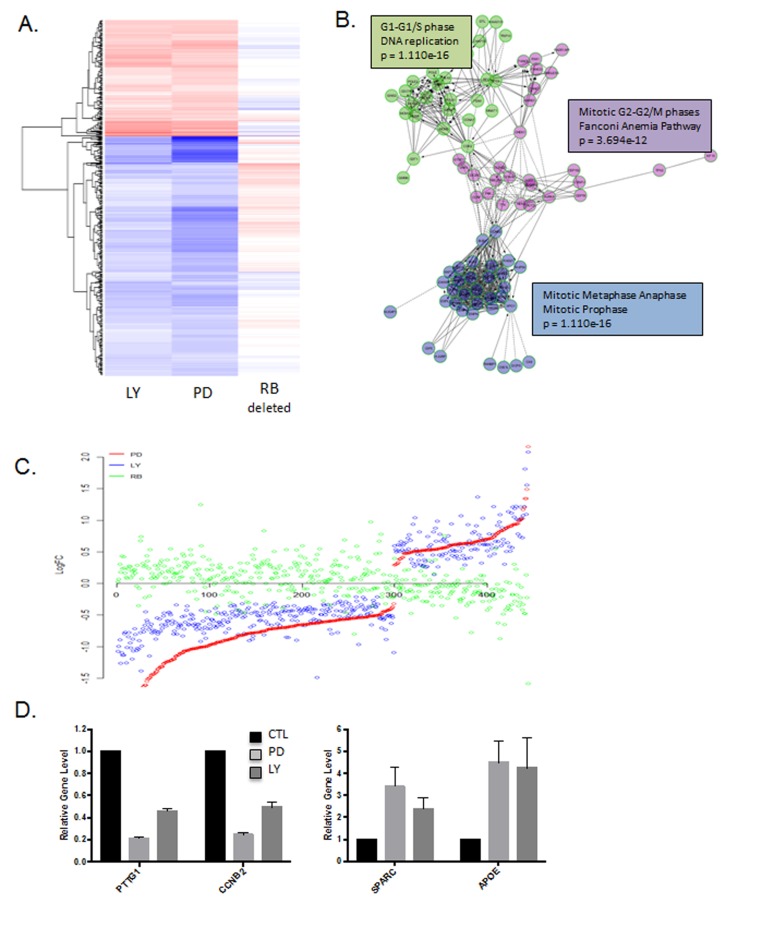
Gene expression responses to CDK4/6 inhibition ***in vivo***: **A**. Heatmap depicting genes that were altered by treatment with either abemaciclib (LY) or palbociclib (PD). The expression of these genes in MB231 RB CRISPR/CAS9 deleted tumors is shown for comparison. **B**. Network analysis of genes repressed by abemaciclib demonstrating key enrichment of genes regulating cell cycle transitions. **C**. Comparison of gene expression changes in LY, PD and RB deleted cells. Log fold change is plotted on the y-axis with a given gene indicated by the dot. **D**. Select genes that are repressed or induced by abemaciclib (LY) and palbociclib (PD).

To more rigorously evaluate the molecular features of response to palbociclib vs. abemaciclib, tumor tissue was triaged for gene expression analysis. As shown in the heatmap, there was almost universal concordance in the transcriptional response to palbociclib and abemaciclib (Figure 6A and 6B, Supplementary Figure 3). The transcriptional repression was dominated by cell cycle associated genes, similar to findings from cell culture models (Figure 6C, Supplementary Figure 3). Interestingly, the up-regulated genes were also well conserved in response to palbociclib and abemaciclib (Figure 6C and 6D, Supplementary Figure 3), but represented a diverse collection of ontologies that were not significantly enriched for a specific biological funcion. These data illustrate that in the *in vivo* setting the response to these structurally diverse agents is remarkably similar.

**Figure 7:**
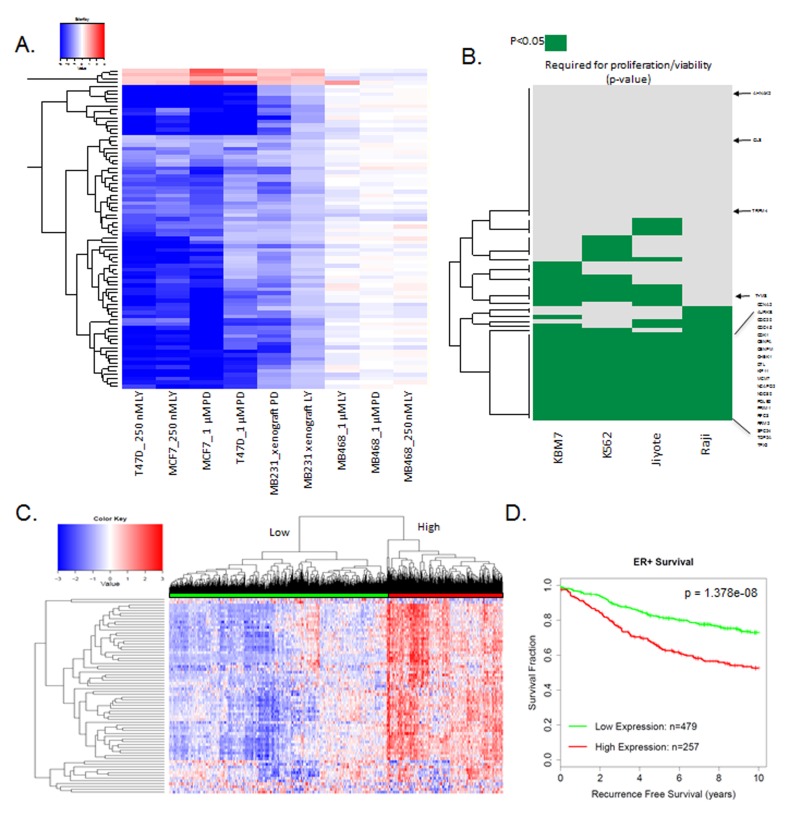
Composite response signature for CDK4/6 inhibitors **A**. Heatmap showing genes that are consistently altered with palbociclib (PD) and abemacicilb (LY) in RB-proficient models. RB-deficient MB468 model is shown as a control. **B**. Data plotting genes within the CDK4/6 response signature that are required for viability (*p* < 0.05) for the indicated cell lines. The CDK4/6 response signature genes are strongly associated with reduced viability/proliferation across all of the cell lines interrogated (two-sided test of equal proportions, *p* = 4.231703e-17). Select required genes are shown for reference **C**. Heatmap showing the relative expression of the CDK4/6 response signature across 967 ER+ breast cancer specimens. Two major clusters are identified. **D**. Kaplan-Meier curve dichotomized by the two major clusters observed in ER+ breast cancer specimens (log-rank p-value=1.378e-08).

The findings *in vivo* support the development of a response marker for CDK4/6 inhibition that is applicable to all agents in clinical development. Gene expression data with palbociclib and abemaciclib in MCF7 and T47D cell lines as well as MB231 xenograft tumors were employed to develop a composite signature for response to CDK4/6 inhibition. Specifically, genes that were changed by greater than 1.5-fold in the same direction in all conditions were selected (Figure 7A). A total of 78 genes were consistently repressed across all conditions with both agents and only 4 genes were consistently upregulated. The induced genes have diverse, but important function in tumor biology, including GLS which is a rate limiting determinant of glutamine metabolism. As expected, repressed genes included multiple cell cycle regulated genes; however, they also encompassed a number of genes associated with DNA damage repair/response (BARD1, FANCE) and signaling cascades (e.g., TACC3, RANBP1). As a means to evaluate the significance of these genes, we queried their relationship to genes that are required for the proliferation and viability of human cell lines [21]. Of the genes in the composite CDK4/6 response signature, 20 were associated with broad inhibition of viability representing a significant enrichment (two-sided test of equal proportions, the p-value is 4.231703e-17) for essential viability genes (Figure 7B). As shown in the heatmap, both well-known cell cycle target genes (e.g., MCM2 and CDK1) as well as poorly characterized CDK4/6 target genes (e.g., DTL and TPX2) are required for cellular survival and proliferation. Together, these data suggest that the repression of these genes is likely critical for the functional activity of CDK4/6 inhibitors. Applying the CDK4/6 response signature across ER-positive breast cancer demonstrated two distinct subtypes (Figure 7C). The subtype which expressed generally higher levels of the CDK4/6 response signature genes was strongly associated with prognosis, and suggests that the suppression of these critical target genes contributes to the activity in ER-positive breast cancers (Figure 7D).

## DISCUSSION

Many CDK inhibitory drugs have been developed, but only recently specific CDK4/6 inhibitors demonstrated efficacy in the clinic [1, 2, 22, 23]. The existing CDK4/6 inhibitors diverge into two clades based on dosing schedules and toxicity profile [1, 3, 7]. Here, we used a combination of molecular and functional approaches to delineate the biological features of structurally disparate CDK4/6 inhibitors.

Since cell cycle pathways have been well-defined, we initially explored the canonical feature of CDK4/6 inhibitors on cell cycle [4]. Both abemaciclib and palbociclib inhibited cell cycle progression with a 2N DNA-content, and the observed arrest was dependent on the presence of the RB-tumor suppressor. Additionally, both abemaciclib and palbociclib suppress a cadre of E2F-regulated genes that are highly conserved. These repressed genes contribute to the biological differences between luminal A and luminal B subtypes of breast cancer and are associated with prognosis of ER+ breast cancer [18, 24]. Consistent with earlier work published with palbociclib [18], the expression of genes induced by the treatment with CDK4/6 inhibitors is more variable across tumor types and models. These adaptive responses to CDK4/6 inhibition could be important in defining durability of response to CDK4/6 inhibition [4, 25, 26]. *In vivo* studies demonstrated that gene-expression changes following treatment with palbociclib or abemaciclib were remarkably similar. By employing the totality of the data we were able to develop a composite signature for CDK4/6 inhibition that was common to palbociclib or abemaciclib response across all models tested. Interestingly, this signature has both well-known cell cycle regulated genes and a number of new target genes that contribute to diverse cellular processes. Genes encompassed in this signature are highly-enriched as required for cellular proliferation and viability, suggesting that suppression of this cadre of genes is critical for the activity of CDK4/6 inhibitors and could be employed as pharmacodynamic markers.

While the CDK4/6 inhibitors exhibited important similarities relevant to cell cycle inhibition, there were several important distinctions. Palbociclb elicits minimal cellular toxicity up to doses of 5 µM; however, abemaciclib can demonstrate cellular toxicity at 250 nM depending on the cell type. The cytotoxic effect of abemaciclib is RB independent and ostensibly reflects an off-target effect of the agent. This finding is consistent with recently reported data that abemaciclib has broader kinase inhibitory activity relative to palbociclib or ribociclib [13]. The phenotype associated with cytotoxicity driven by abemaciclib is linked to the appearance of a multi-vacuolar phenotype. This phenotype has not been previously reported for either palbociclib or ribociclib, suggesting that this represents a specific off-target feature of abemaciclib. Interestingly, in RB-deficient cells abemaciclib could elicit changes in gene expression that were distinct from the transcriptional repression of cell cycle and included a number of genes that are controlled by casein kinase [19]. The inhibition of casein kinase, DYRK, and HIPK family members specific to abemaciclib was likely triggering multi-vacuolar phenotype as CX-4945, an agent inhibiting these kinases, had similar effect on cell morphology [12, 13]. Together, these data suggest that there are off-target, biologically tractable effects of abemaciclib that imply less specificity relative to palbociclib and indicate the importance of dose relationships with regard to biological effects in preclinical models.

The most clinically significant aspect of these drugs is their behavior when dosed orally in animals. Analysis of palbociclb and abemaciclib showed highly concordant effects on tumor biology in terms of the suppression of cell cycle progression in an RB-dependent fashion, and no evidence for the multi-vacuolar phenotype or cytoxicity in the models employed. Additionally, there were no obvious indications of tissue toxicity in the analysis of multiple organs. Consistent with the clinical experience, while palbociclib induced substantial reduction in neutrophil counts, abemaciclib had a more modest effect at the employed doses. However, molecular analysis of the tumors treated with palbociclib or abemaciclib demonstrated that they exhibited veritably identical changes in gene expression with treatment. These data suggest that with oral dosing the activity of abemaciclib is restricted to the suppression of CDK4/6 and comparable to palbociclib. These data have significant implications relative to biomarkers and pharmacodynamic targets, as it would suggest that findings from one class of CDK4/6 inhibitor largely hold true across all classes of selective CDK4/6 targeting drugs. This point is illustrated through the development of a composite signature of response to CDK4/6 inhibitors. Additionally, these data would suggest that there is little rationale for treating tumors with different CDK4/6 inhibitors concurrently, as the fundamental mechanisms of action and resistance is consistent across the structurally diverse inhibitors.

## MATERIALS AND METHODS

### Cells and drug treatments

Established cell lines (BT549, MDA-MB-231, MDA-MB-468, MCF7, T47D, and AW23) were cultured in DMEM +10% FBS. Cells were treated with PD-0332991 (PD-palbociclib), LY235219 (LY-abemaciclib), chloroquine (CQ), CX-4945, and dinaciclib. Abemaciclib was provided by Eli Lilly. The other agents were purchased from Sellek-Chem.

### CRISPR gene editing

Established cell lines (MDA-MB-231, MCF7, T47D) were transfected with a plasmid containing a RB-specific guide RNA sequence, Cas9 protein, and GFP using Lipofectamine 3000 transfection reagent. Three days after transfection, single GFP+ cells were sorted into 96-well plates and expanded. Following expansion, loss of RB expression was confirmed by immunofluorescence screening and western blot.

### Acridine orange staining

Cells were seeded in tissue culture plates. Cells either pre-treated with chloroquine or for 2 hours or left untreated were treated with LY235219. At the indicated time points cells were loaded with acridine orange (2µM) and incubated at 37°C for 30 minutes. Cells were washed twice in PBS+3% FBS and imaged immediately on an EVOS FL Cell Imaging System with an excitation of 488nm and emission at both 510nm and 624nm. Staining indicates lysosomal membrane permeability by indicating the acridine concentration gradient from red (high concentration sequestered in lysosomes) to green (low concentration).

### Proliferation and cell number quantitation

Cells were plated overnight with or without LY235219 or PD-0332991. Proliferation was determined using a chemiluminescent BrdU ELISA (Roche, catalog #11669915001). Cells were incubated with BrdU labeling reagent for 2 hours and processed as described by the manufacturer. Luminescence was read on a Biotek Synergy 2 plate reader. Parallel experiments were performed as using flow-cytometry as previously published. To account for differences in cell counts, cytotoxicity and proliferation measurements were normalized to cell number as determined by Cell Titer Glo assay. Cell Titer Glo reagent was added to each well in fresh PBS and cells were incubated for 10 minutes at room temperature. Luminescence was read on a BioTek Synergy 2 plate reader.

### Mice and mammary xenografts

Female NOD/SCID mice were maintained in the UT Southwestern Medical Center animal facility. All mouse care, treatment, and sacrifice was approved by the UT Southwestern Institutional Animal Care and Use Committee (IACUC) in accordance with the National Institutes of Health (NIH) Guide for the Care and Use of Laboratory Animals. At 6-8 weeks of age, animals were surgically manipulated under anesthesia to reveal the mammary gland. Each mouse was injected with 1×10^6^ of the indicated cells in the mammary gland. Mice were monitored for tumor formation until palpable tumors were detected. Mice were randomized based on tumor size and either treated daily with PD-0332991 (palbociclib, 125mg/kg) or LY2835219 (abemaciclib, 100mg/kg) for 7 days by oral gavage, or left untreated. One day after the final administration, mice were sacrificed and tumors, blood, and other tissues were collected for analysis.

### Pathological evaluation

Isolated tumor samples were fixed in 10% neutral buffered formalin for 48-72 hours, processed, and paraffin embedded. Specimens were cut to a thickness of 4mm and stained with hematoxylin and eosin or Ki67 as previously described [27, 28].

### Blood counts

Prior to the administration of CDK4/6 inhibitors and at the completion of the study blood was drawn from the submandibular vein by 4mm lancet and collected in EDTA-coated collection tubes to prevent coagulation. Complete blood counts were acquired on a ProCyte DX hematology analyzer (IDEXX Laboratories, Westbrook, ME, USA).

### Gene expression array and analysis

Following treatment with LY235219 or PD-0332991 cell were collected and RNA was isolated using a Qiagen RNeasy kit. RNA was isolated from snap frozen tumor tissue. Microarray analysis was performed using standard approaches. Log fold-changes were calculated from quantile-normalized microarray data and p-values determined using a two-tailed Student's homoscedastic t-test. Genes with a p-value greater than 0.05 in RB positive samples were excluded from further analysis. In the MB468 comparison heatmap (Figure 3B), genes with a p-value greater than 0.05 in LY235219 treated samples were excluded from further analysis. The CDK4/6 inhibitor response signature was determined by filtering all cell line and in vivo RB-positive treatments for genes with log fold-changes more extreme than +/-0.5 that are consistently up- or down-regulated across all conditions. P-values determining which genes are essential for cell viability were obtained from Wang et. al. 2015 [21]. A gene was labeled as “essential” if p-values were significant in the tested cell lines (*p* < 0.05). A two-sided test of equal proportions was performed to compare the proportion of essential genes in the gene signature to the proportion of essential genes in the entire gene set. Unsupervised hierarchical clustering was performed using R. Network diagrams were generated and analyzed using ReactomeFIViz ^1^. Scatterplots of log fold-changes were generated, with values from a single case determining gene order (i.e. x-axis). Downregulated genes in treated cell lines were reassessed using microarray data from 967 clinical ER-positive breast cancer cases [18]. Cases were ordered based on the average expression of the downregulated gene set and gene-wise hierarchical clustering was performed. Low/high expression groups were defined using the median of the average expression scores. Unsupervised hierarchical clustering was performed for both genes and samples using the CDK4/6 gene signature and low/high expression was determined based on the corresponding dendogram branch. Cases missing survival and/or relapse data were excluded. Kaplan-Meier curves were generated for recurrence-free survival using the “survival” R package and p-values determined using the Cox proportional hazards model. Data is available on line (Supplementary Data Files) and raw data has been deposited in GEO under the title of this manuscript.

## SUPPLEMENTARY MATERIALS FIGURES AND TABLES








