# Supplementary material for: Biological specificity of CDK4/6 inhibitors: dose response relationship, in vivo signaling, and composite response signature
Source: Oncotarget. 2017 Jun 10;8(27):43678–91. doi: 10.18632/oncotarget.18435 (PMC5546433; doi:10.18632/oncotarget.18435)
Supplement: Supplementary file 1 [file oncotarget-08-43678-s001.pdf]

Biological specificity of CDK4/6 inhibitors: dose response relationship, *in vivo* signaling, and composite response signature

Supplementary Material

Genes where p < 0.1 for MCF7 and T47D for both PD and LY (n = 599).

	MCF7		T47D		MCF7/T47D Comparison		
	Upregulated	Downregulated	Upregulated	Downregulated	Upregulated Concordance	Downregulated Concordance	Overall Concordance
PD	221	378	271	328	73.24%	80.56%	87.31%
LY	231	368	238	361	90.63%	93.88%	96.16%
Concordance (PD/LY)	82.26%	88.86%	69.10%	76.21%			
Overall Concordance (PD/LY)	92.65%		84.47%				

LY downregulated

GO biological process complete	GO Term	Homo sapiens - REFLIST (20972)	upload_1 (550)	upload_1 (expected)	upload_1 (over/under)	upload_1 (fold Enrichment)	upload_1 (P-value)
cell cycle	GO:0007049	1337	272	46.41	+	5.86	1.47E-127
cell cycle process	GO:0022402	1091	244	37.87	+	6.44	4.19E-121
mitotic cell cycle	GO:0000278	794	209	27.56	+	7.58	1.48E-114
mitotic cell cycle process	GO:1903047	766	205	26.59	+	7.71	1.99E-113
nuclear division	GO:0000280	518	150	17.98	+	8.34	9.34E-85
organelle fission	GO:0048285	549	151	19.06	+	7.92	2.46E-82
mitotic nuclear division	GO:0007067	385	126	13.36	+	9.43	4.08E-76
chromosome organization	GO:0051276	1012	186	35.13	+	5.29	4.88E-75
DNA metabolic process	GO:0006259	781	159	27.11	+	5.86	7.85E-69
cell division	GO:0051301	476	128	16.52	+	7.75	1.42E-67

LY upregulated

GO biological process complete	GO Term	Homo sapiens - REFLIST (20972)	upload_1 (550)	upload_1 (expected)	upload_1 (over/under)	upload_1 (fold Enrichment)	upload_1 (P-value)
G-protein coupled receptor signaling pathwa	GO:0007186	1193	3	31.29	-	< 0.2	5.38E-07
small molecule metabolic process	GO:0044281	1768	92	46.37	+	1.98	2.22E-06
organonitrogen compound metabolic proces	GO:1901564	1835	91	48.12	+	1.89	2.99E-05
organic substance catabolic process	GO:1901575	1594	79	41.8	+	1.89	3.92E-04
catabolic process	GO:0009056	1886	89	49.46	+	1.8	4.44E-04
single-organism metabolic process	GO:0044710	3544	142	92.94	+	1.53	7.14E-04
cellular catabolic process	GO:0044248	1581	77	41.46	+	1.86	1.19E-03
organic acid metabolic process	GO:0006082	956	54	25.07	+	2.15	1.34E-03
oxoacid metabolic process	GO:0043436	939	53	24.63	+	2.15	1.80E-03
monocarboxylic acid metabolic process	GO:0032787	508	35	13.32	+	2.63	3.04E-03
carboxylic acid metabolic process	GO:0019752	832	48	21.82	+	2.2	3.77E-03

Supplementary Figure 1: Genes where p < 0.1 for MCF7 and T47D for both PD and LY (n = 599).

Genes where p < 0.1 for MCF7 and T47D for both PD and LY (n = 599).

	MB468 Comparison	
	Overall Concordance w/MCF7	Overall Concordance w/T47D
PD	54.59%	49.25%
LY (250uM LY)	53.92%	53.09%

LY upregulated

GO biological process complete	GO Term	Homo sapiens - REFLIST (20972)	upload_1 (463)	upload_1 (expected)	upload_1 (over/under)	upload_1 (fold Enrichment)	upload_1 (P-value)
ncRNA processing	GO:0034470	392	33	8.65	+	3.81	9.80E-07
ncRNA metabolic process	GO:0034660	545	39	12.03	+	3.24	2.07E-06
metabolic process	GO:0008152	9790	274	216.15	+	1.27	3.77E-04
ribosome biogenesis	GO:0042254	317	25	7	+	3.57	6.20E-04
rRNA processing	GO:0006364	257	22	5.67	+	3.88	9.62E-04
rRNA metabolic process	GO:0016072	282	23	6.23	+	3.69	1.16E-03
RNA processing	GO:0006396	868	45	19.16	+	2.35	1.34E-03
mitochondrial transport	GO:0006839	229	20	5.06	+	3.96	2.88E-03
cellular metabolic process	GO:0044237	8825	248	194.79	+	1.27	3.48E-03
single-organism metabolic process	GO:0044710	3544	119	78.24	+	1.52	9.33E-03
ribosomal large subunit biogenesis	GO:0042273	61	10	1.35	+	7.45	1.22E-02

LY downregulated

GO biological process complete	GO Term	Homo sapiens - REFLIST (20972)	upload_1 (739)	upload_1 (expected)	upload_1 (over/under)	upload_1 (fold Enrichment)	upload_1 (P-value)
biological regulation	GO:0065007	11693	508	412.05	+	1.23	2.76E-09
regulation of cellular process	GO:0050794	10463	462	368.69	+	1.25	2.77E-08
regulation of biological process	GO:0050789	11075	478	390.25	+	1.22	3.37E-07
positive regulation of cellular process	GO:0048522	4774	245	168.22	+	1.46	6.04E-07
negative regulation of biological process	GO:0048519	4737	241	166.92	+	1.44	2.26E-06
positive regulation of biological process	GO:0048518	5321	262	187.5	+	1.4	6.40E-06
negative regulation of cellular process	GO:0048523	4261	217	150.15	+	1.45	2.74E-05
biological_process	GO:0008150	17240	663	607.49	+	1.09	6.91E-05
regulation of metabolic process	GO:0019222	6380	293	224.81	+	1.3	3.03E-04
regulation of cellular metabolic process	GO:0031323	5930	276	208.96	+	1.32	6.78E-04
regulation of macromolecule metabolic process	GO:0060255	5910	274	208.25	+	1.32	8.28E-04
positive regulation of cellular metabolic process	GO:0031325	2901	154	102.22	+	1.51	1.02E-03
positive regulation of metabolic process	GO:0009893	3095	162	109.06	+	1.49	1.06E-03

Supplementary Figure 2: Genes where p < 0.1 for MCF7 and T47D for both PD and LY (n = 599).

In vivo - p < 0.1 for both PD and LY (n = 2109)

	In vivo	
	Upregulated	Downregulated
PD	914	1195
LY	916	1193
Concordance (PD/LY)	93.86%	95.26%
Overall Concordance (PD/LY)	97.25%	

LY/PD downregulated

GO biological process complete	GO Term	Homo sapiens - REFLIST (20972)	upload_1 (540)	upload_1 (expected)	upload_1 (over/under)	upload_1 (fold Enrichment)	upload_1 (P-value)
cell cycle	GO:0007049	1337	177	34.43	+	5.14	8.07E-72
mitotic cell cycle	GO:0000278	794	137	20.44	+	6.7	6.96E-67
cell cycle process	GO:0022402	1091	156	28.09	+	5.55	2.25E-66
mitotic cell cycle process	GO:1903047	766	134	19.72	+	6.79	6.18E-66
chromosome organization	GO:0051276	1012	129	26.06	+	4.95	7.60E-48
mitotic nuclear division	GO:0007067	385	84	9.91	+	8.47	3.10E-46
nuclear division	GO:0000280	518	93	13.34	+	6.97	1.07E-44
organelle fission	GO:0048285	549	95	14.14	+	6.72	1.95E-44
DNA metabolic process	GO:0006259	781	107	20.11	+	5.32	2.15E-41
chromosome segregation	GO:0007059	295	70	7.6	+	9.22	3.94E-40
organelle organization	GO:0006996	3186	213	82.04	+	2.6	1.48E-38
cell division	GO:0051301	476	82	12.26	+	6.69	1.44E-37
DNA replication	GO:0006260	217	59	5.59	+	10.56	1.97E-36
nuclear chromosome segregation	GO:0098813	254	62	6.54	+	9.48	8.19E-36
sister chromatid segregation	GO:0000819	183	53	4.71	+	11.25	9.98E-34
cell cycle phase transition	GO:0044770	268	60	6.9	+	8.69	1.64E-32
mitotic cell cycle phase transition	GO:0044772	262	59	6.75	+	8.75	4.76E-32

LY/PD upregulated

GO biological process complete	GO Term	Homo sapiens - REFLIST (20972)	upload_1 (303)	upload_1 (expected)	upload_1 (over/under)	upload_1 (fold Enrichment)	upload_1 (P-value)
single-organism cellular process	GO:0044763	9816	190	141.82	+	1.34	1.57E-04
single-organism metabolic process	GO:0044710	3544	89	51.2	+	1.74	4.41E-04
organophosphate biosynthetic process	GO:0090407	496	23	7.17	+	3.21	1.08E-02
carbohydrate derivative biosynthetic process	GO:1901137	596	25	8.61	+	2.9	2.17E-02
basement membrane organization	GO:0071711	15	5	0.22	+	23.07	2.65E-02
lipid metabolic process	GO:0006629	1234	39	17.83	+	2.19	3.64E-02

Supplementary Figure 3: *In vivo*- p < 0.1 for both PD and LY (n = 2109) .
